# Supplementary material for: Flexible DMRG-Based Framework for Anharmonic Vibrational Calculations
Source: J Chem Theory Comput. 2023 Dec 7;19(24):9329–43. doi: 10.1021/acs.jctc.3c00902 (PMC10753801; doi:10.1021/acs.jctc.3c00902)
Supplement: Supplementary file 1 — ct3c00902_si_001.pdf [file ct3c00902_si_001.pdf]

Supporting Information: Flexible DMRG-based framework for anharmonic vibrational calculations

Nina Glaser, Alberto Baiardi, and Markus Reiher*

ETH Zurich, Department of Chemistry and Applied Biosciences, Vladimir-Prelog-Weg 2, 8093 Zurich, Switzerland

E-mail: mreiher@ethz.ch

1 Fourier-DVR Matrix Elements

In the DVR with a uniform grid, the potential $\mathcal{V}$ entering the VSCF Hamiltonian can be straight-forwardly evaluated with

$$\mathcal{V}_{\mu\omega} = \mathcal{V}(Q_\mu)\delta_{\mu\omega} \, , \tag{1}$$

where Q_μ is a point on the multidimensional grid used both in the PES construction and the DVR-VSCF. The kinetic energy can be computed for $\mu \neq \omega$ with[S1]

$$\mathcal{T}_{\mu\omega} = \frac{\pi^2(-1)^{(\mu-\omega)}}{4R^2} \left[\frac{1}{\sin^2\left(\frac{\pi(\mu-\omega)}{2(N_\mathrm{P}+1)}\right)} - \frac{1}{\sin^2\left(\frac{\pi(\mu+\omega+2)}{2(N_\mathrm{P}+1)}\right)} \right] \, , \tag{2}$$

where we define $R = |2(Q_{\mathrm{max}} + \Delta Q)|$, and N_{P} is the number of primitve Fourier basis functions. For $\mu = \mu$, the kinetic term simplifies to

$$\mathcal{T}_{\mu\mu} = \frac{\pi^2}{4R^2}\left[\frac{2\left(N_{\mathrm{P}}+1\right)^2+1}{3} - \frac{1}{\sin^2\left(\frac{\pi(\mu+1)}{N_{\mathrm{P}}+1}\right)}\right] . \tag{3}$$

2 Convergence Analysis of Vibrational Structure Calculation Parameters on Water

For a convergence analysis of the calculation parameters, several PESs of water are constructed with different settings, and VSCF, VCI, and vDMRG calculations are performed. For the electronic structure single points, CCSD(T)-F12 calculations[S2] with an cc-pVTZ-F12 basis set[S3] are performed with the Orca program.[S4]

Table S1: Comparison of harmonic, VSCF, VCI, and vDMRG energies for the fundamental excitations of water for different PES construction settings. All energies are given in cm^{-1} and the fundamental excitation energies are given relative to the corresponding zero-point vibrational energy (ZPVE). The default settings for the PES construction are a mode-coupling order of $n = 3$, $N_P = 11$ primitive basis functions, and the $N_\nu = 5$ harmonic inversion point. PES mod. denotes the modification of these settings from their default value. For water, a mode coupling order of 3 results in the full-dimensional PES, and VCI(3,15) corresponds to full CI in the CI space of 6 modals per mode.

State	Harm.	PES mod.	VSCF	VCI(3,15)	FEAST-S	FEAST-L	ORTHO-L
ZPVE	4714.4	-	4669.2	4650.1	4650.1	4650.1	4650.1
		$n = 1$	4725.6	4725.6	4725.6	4725.6	4725.6
		$n = 2$	4668.1	4648.0	4648.0	4648.0	4648.0
		$N_\nu = 3$	4672.4	4653.9	4653.9	4653.9	4653.9
		$N_\nu = 7$	4669.2	4650.1	4650.1	4650.1	4650.1
		$N_P = 7$	4660.5	4640.3	4640.3	4640.3	4640.3
		$N_P = 15$	4669.3	4650.3	4650.3	4650.3	4650.3
Bend	1650.8	-	1589.1	1584.9	1584.9	1584.9	1584.9
		$n = 1$	1635.9	1635.9	1635.9	1635.9	1635.9
		$n = 2$	1588.6	1582.2	1582.2	1582.2	1582.2
		$N_\nu = 3$	1594.7	1591.1	1591.1	1591.1	1591.1
		$N_\nu = 7$	1589.0	1584.8	1584.8	1584.8	1584.8
		$N_P = 7$	1606.3	1602.8	1602.8	1602.8	1602.8
		$N_P = 15$	1589.2	1585.1	1585.1	1585.1	1585.1
Symmetric stretch	3834.0	-	3717.1	3660.5	3660.5	3660.5	3660.5
		$n = 1$	3750.2	3750.2	3750.2	3750.2	3750.2
		$n = 2$	3717.4	3651.7	3651.7	3651.7	3651.7
		$N_\nu = 3$	3761.2	3709.3	3709.3	3709.3	3709.3
		$N_\nu = 7$	3715.4	3658.6	3658.6	3658.6	3658.6
		$N_P = 7$	3750.8	3680.6	3680.6	3680.6	3680.6
		$N_P = 15$	3719.6	3663.2	3663.2	3663.2	3663.2
Asymmetric stretch	3944.0	-	3778.4	3742.9	3743.0	3743.0	3743.0
		$n = 1$	4037.9	4037.9	4037.9	4037.9	4037.9
		$n = 2$	3762.2	3722.6	3722.8	3722.8	3722.8
		$N_\nu = 3$	3794.6	3765.6	3765.6	3765.6	3765.6
		$N_\nu = 7$	3778.2	3742.5	3742.6	3742.6	3742.6
		$N_P = 7$	3805.8	3780.5	3780.5	3780.5	3780.5
		$N_P = 15$	3778.8	3743.7	3743.8	3743.8	3743.8

3 Additional Vibrational Structure Calculation Results for Methyloxirane

Table S2: Comparison of harmonic, VSCF, VCI and vDMRG energies for the fundamental excitations of methyloxirane ordered according to their harmonic energy. The potential energy surface considered is a two-body on-the-fly calculated PES which only treats the fingerprint region as coupled. Mode 1 and modes 19-24 are decoupled, as highlighted by the star *. All energies are given in cm^{-1} and the fundamental excitation energies are given relative to the corresponding zero-point vibrational energy (ZPVE).

State	Harmonic	DVR-VSCF	VCI(2,10)	FEAST-S	FEAST-L	ORTHO-L
ZPVE	18586.2	18814.9	18811.1	18811.7	18811.1	18811.1
$\nu_1^\star$	194.7	381.4	-	-	-	-
ν_2	366.4	406.3	405.4	405.4	405.4	405.4
ν_3	411.3	453.7	452.8	453.0	452.8	452.8
ν_4	760.5	770.6	767.1	767.4	767.1	767.1
ν_5	837.0	842.0	838.9	839.0	838.8	838.8
ν_6	894.2	960.8	956.6	955.3	955.1	955.6
ν_7	964.1	978.2	981.2	980.1	980.0	980.2
ν_8	1026.0	1072.3	1071.3	1071.3	1071.1	1071.2
ν_9	1110.0	1149.5	1148.4	1148.5	1148.3	1148.2
ν_{10}	1139.2	1193.0	1191.2	1190.4	1190.3	1190.2
ν_{11}	1150.7	1180.6	1178.0	1177.8	1177.6	1177.5
ν_{12}	1175.3	1199.8	1204.3	1202.7	1202.6	1202.6
ν_{13}	1282.8	1295.6	1293.0	1293.1	1292.8	1292.7
ν_{14}	1381.5	1413.4	1412.8	1413.6	1413.7	1412.7
ν_{15}	1427.5	1441.2	1439.7	1440.7	1440.7	1439.6
ν_{16}	1454.3	1467.0	1466.8	1467.2	1466.9	1466.8
ν_{17}	1471.2	1483.6	1483.1	1483.4	1483.3	1482.9
ν_{18}	1516.8	1533.2	1527.2	1521.0	1520.9	1520.9
$\nu_{19}^\star$	3028.6	2990.7	-	-	-	-
$\nu_{20}^\star$	3085.5	3027.5	-	-	-	-
$\nu_{21}^\star$	3096.7	3164.7	-	-	-	-
$\nu_{22}^\star$	3099.8	3058.1	-	-	-	-
$\nu_{23}^\star$	3120.3	3166.2	-	-	-	-
$\nu_{24}^\star$	3177.8	3246.5	-	-	-	-

Table S3: Comparison of harmonic, VSCF, VCI, and vDMRG energies for the fundamental excitations of methyloxirane ordered according to their harmonic energy. The potential energy surface considered is a two-body on-the-fly calculated PES with the first mode decoupled, as highlighted by the star *. The C-H strechings marked by † could not be uniquely assigned, as there is no single dominating CI coefficient of those fundamental in the calculated states. All energies are given in cm^{-1} and the fundamental excitation energies are given relative to the corresponding zero-point vibrational energy (ZPVE).

State	Harmonic	DVR-VSCF	VCI(2,10)	FEAST-S	FEAST-L	ORTHO-L
ZPVE	18586.2	18482.7	18421.0	18422.7	18419.4	18419.4
$\nu_1^\star$	194.7	381.4	-	-	-	-
ν_2	366.4	376.4	372.8	372.9	372.6	372.6
ν_3	411.3	423.7	419.7	419.4	419.5	419.5
ν_4	760.5	753.8	749.0	749.6	748.5	749.4
ν_5	837.0	826.5	822.2	824.2	821.9	821.7
ν_6	894.2	901.5	898.7	895.5	894.4	894.4
ν_7	964.1	955.7	946.8	953.8	952.9	952.9
ν_8	1026.0	1021.3	1014.6	1018.0	1017.5	1017.4
ν_9	1110.0	1102.8	1101.3	1099.1	1098.1	1097.4
ν_{10}	1139.2	1134.4	1126.8	1126.0	1125.9	1126.2
ν_{11}	1150.7	1139.2	1135.8	1136.3	1135.4	1135.4
ν_{12}	1175.3	1163.1	1158.6	1164.7	1163.1	1163.1
ν_{13}	1282.8	1267.3	1264.2	1264.4	1262.4	1262.7
ν_{14}	1381.5	1365.5	1363.1	1362.9	1362.3	1362.6
ν_{15}	1427.5	1401.7	1399.1	1402.3	1398.1	1398.1
ν_{16}	1454.3	1419.6	1417.4	1417.9	1417.2	1417.2
ν_{17}	1471.2	1437.9	1435.6	1435.9	1435.1	1435.1
ν_{18}	1516.8	1490.3	1484.4	1485.3	1476.8	1476.8
ν_{19}	3028.6	2925.2	†	†	†	-
ν_{20}	3085.5	2951.3	2893.6	2890.2	2884.2	-
ν_{21}	3096.7	2900.3	2833.8	2883.6	2872.5	-
ν_{22}	3099.8	2944.4	2943.5	†	2921.5	-
ν_{23}	3120.3	2958.1	2820.5	†	2987.7	-
ν_{24}	3177.8	2966.8	2931.1	2933.2	2932.8	-

4 Example Inputs for n-mode vDMRG Calculations with QCMaquis

Example input settings for a ground state n-mode vDMRG calculation with QCMaquis:

```
max_bond_dimension           = 50
nsweeps                      = 50
ngrowsweeps                  = 10
nmainsweeps                  = 20
alpha_initial                = 1.0E-8
alpha_main                   = 1.0E-10
alpha_final                  = 0
truncation_initial           = 0
truncation_final             = 0
eigensolver                  = IETL_JCD
optimization                 = singlesite
integral_file                = pes_mc2.txt
integral_cutoff              = 1.0E-8
resultfile                   = mo_result_gs.h5
chkpfile                     = mo_chkp_gs
symmetry                     = nu1
MODEL                        = nmode
LATTICE                      = "nmode lattice"
L                            = 144
nmode_num_modes              = 24
nmode_num_basis              = "6,6,6,6,6,6,6,6,6,6,6,6,6,6,6,6,6,6,6,6,6,6,6,6"
init_type                    = basis_state_generic
init_basis_state             = "0,0,0,0,0,0,0,0,0,0,0,0,0,0,0,0,0,0,0,0,0,0,0,0"
```

Example input settings for an excited state n-mode vDMRG[FEAST] calculation with QCMaquis targeting the fundamental excitations ν_1, ν_2, and ν_3 simultaneously:

```
feast_emin                    = 18550
feast_emax                    = 18850
feast_num_states              = 4
feast_max_iter                = 5
max_bond_dimension            = 50
nsweeps                       = 50
ngrowsweeps                   = 10
nmainsweeps                   = 20
alpha_initial                 = 1.0E-8
alpha_main                    = 1.0E-10
alpha_final                   = 0
truncation_initial            = 0
truncation_final              = 0
eigensolver                   = IETL_JCD
optimization                  = singlesite
integral_file                 = pes_mc2.txt
integral_cutoff               = 1.0E-8
resultfile                    = mo_result.h5
chkpfile                      = mo_chkp
symmetry                      = nu1
MODEL                         = nmode
LATTICE                       = "nmode lattice"
L                             = 144
nmode_num_modes               = 24
nmode_num_basis               = "6,6,6,6,6,6,6,6,6,6,6,6,6,6,6,6,6,6,6,6,6,6,6,6"
init_type                     = basis_state_generic
init_basis_state              = "1,0,0,0,0,0,0,0,0,0,0,0,0,0,0,0,0,0,0,0,0,0,0,0|
                                 0,1,0,0,0,0,0,0,0,0,0,0,0,0,0,0,0,0,0,0,0,0,0,0|
                                 0,0,1,0,0,0,0,0,0,0,0,0,0,0,0,0,0,0,0,0,0,0,0,0"
init_space                    = "2,2,0,0,0,0,0,0,0,0,0,0,0,0,0,0,0,0,0,0,0,0,0,0"
```

References

(S1) Colbert, D. T.; Miller, W. H. A novel discrete variable representation for quantum mechanical reactive scattering via the S-matrix Kohn method. *J. Chem. Phys.* **1992**, *96*, 1982–1991.

(S2) Valeev, E. F.; Daniel Crawford, T. Simple coupled-cluster singles and doubles

method with perturbative inclusion of triples and explicitly correlated geminals: The CCSD(T)R12^{-} model. *The Journal of Chemical Physics* **2008**, *128*, 244113.

(S3) Peterson, K. A.; Adler, T. B.; Werner, H.-J. Systematically convergent basis sets for explicitly correlated wavefunctions: The atoms H, He, B–Ne, and Al–Ar. *J. Chem. Phys.* **2008**, *128*, 084102.

(S4) Neese, F. Software update: The ORCA program system—Version 5.0. *WIREs Comput. Mol. Sci.* **2022**, *12*, e1606.
